# A combined individual and group-based stabilization and skill training intervention versus treatment as usual for patients with long lasting posttraumatic reactions receiving outpatient treatment in specialized mental health care – a study protocol for a randomized controlled trial

**DOI:** 10.1186/s13063-020-04297-z

**Published:** 2020-05-27

**Authors:** K. H. Holgersen, I. Brønstad, M. Jensen, H. Brattland, S. K. Reitan, A. M. Hassel, M. Arentz, M. Lara-Cabrera, A. E. Skjervold

**Affiliations:** 1 Tiller Community Mental Health Centre, Department of Mental Health, Tiller DPS, St. Olavs Hospital HF, Postboks 3250, Torgarden, 7006 Trondheim, Norway; 2 Department of Mental Health (IPH), Faculty of Medicine and Health Sciences, NTNU, Trondheim, Norway

**Keywords:** Community mental health centre, Complex PTSD, Group therapy, Psychoeducation, PTSD, Recovery, Stress-related disorder

## Abstract

**Background:**

Suffering linked to previous interpersonal trauma is common among patients in mental health care. Diagnostic labels may vary, but the clinical picture is often characterized by long-lasting and complex psychological and somatic symptoms, subjective distress and reduced quality of health and life. A substantial proportion of patients do not recover after individual treatment in ordinary specialized mental healthcare settings, despite the proven usefulness of individual trauma-specific treatments. The therapeutic factors that arise in group settings, such as normalization, shame reduction and corrective relational experiences, may be particularly useful for trauma survivors. However, evidence in support of group treatment for trauma survivors is scarce. This study aims to test whether combining a novel group intervention to individual treatment is superior to conventional individual out-patient treatment in an ordinary community mental health hospital.

**Methods:**

In a single-site, non-blinded, randomized controlled trial (RCT), the effect of a combined group-based stabilization and skill-training (SST) intervention added to individual treatment will be compared to conventional treatment (treatment as usual, TAU) alone. Participants (*N* = 160) with ongoing and long-lasting reactions related to known adverse life events from the past will be recruited among patients at general outpatient clinics in a community mental health centre at St. Olav’s University Hospital, Trondheim, Norway. Following baseline assessment and randomization, participants will complete follow-up measures at 4, 8, 13 and 19 months post-baseline. The primary outcome is personal recovery (The questionnaire about the process of recovery , QPR). Secondary outcomes include (1) self-reported symptoms of posttraumatic stress, general mental and somatic health symptoms, well-being, functional impairment and client satisfaction, (2) immunological and endocrine response measured in blood samples and (3) national registry data on occupational status, use of mental health services and pharmacological treatment. Additionally, mechanisms of change via posttraumatic cognitions will be examined.

**Discussion:**

The addition of a group-based intervention to individual treatment for trauma survivors might prove to be an efficient way to meet the need of long-lasting high-intensity treatment in a large group of patients in mental health care, thereby reducing their suffering and increasing their psychosocial functioning.

**Trial registration:**

ClinicalTrials.gov: NCT03887559. Registered on 25 March 2019.

## Background

The rate of exposure to events that are potentially traumatic is high, with estimates in the Western population ranging from 51% to 90% [10, 19, 40]. As demonstrated in longitudinal studies, most people recover after single events [51] but survivors from repeated and interpersonal trauma are more likely to develop long-lasting suffering [16, 35]. Many of these individuals will seek help from mental health services. For instance, the rate of interpersonal trauma among patients in mental health care was reported to be between 62% and 98% in the USA [14] and 71% in Ireland [70]. In Norway, 32–48% of the patients seeking treatment in general outpatient clinics suffered from reactions that were likely related to childhood sexual or physical abuse [27].

The clinical picture of patients exposed to isolated traumatic events is often characterized by re-experiencing the trauma, for example with flashbacks, avoidance of triggering stimuli, and augmented hyperarousal, i.e. the core components of posttraumatic stress disorder (PTSD). A more complex symptom profile is frequently seen among those exposed to repeated interpersonal violence, including emotional dysregulation, a negative self-concept and difficulties in relationships. According to the International Classification of Diseases 11th Revision (ICD-11) these additional symptoms may qualify for a diagnosis of complex PTSD (CPTSD) [71]. But the long-lasting suffering that is associated with the past experiences of trauma survivors often extends beyond a diagnosis of PTSD or CPTSD. These individuals often have other comorbid mental health problems, such as depression, anxiety, substance abuse and interpersonal problems. Recent meta-analyses demonstrate links between early trauma and the development of depression and anxiety, and associations with more profound mental illness have also been documented [25, 44, 45, 63].

Besides affecting mental health, the Adverse Childhood Experience (ACE) study showed in 1998 that people who have undergone trauma have reduced somatic health and increased functional impairment [23]. Severe mental stress in young age, such as neglect or abuse has been shown to increase morbidity and mortality in adult life [24]. Accordingly, childhood abuse and trauma are associated with long-term elevated use of the full-range healthcare system [8]. Somatic disorders that are more frequent in people who have undergone trauma include cardiovascular disease, immune dysfunction and metabolic syndromes [31, 46]. A Danish study found all-cause mortality in a group of people with PTSD to be twice that in a control population [32].

The associations between trauma and somatic health may be mediated by lifestyle factors following the psychological suffering, but there are indications of more direct biological mechanisms as well. First, the neuroendocrine system (like the hypothalamic-pituitary-adrenal (HPA) axis) is clearly involved in long-standing stress, and alterations in thyroid hormones and in cortisol have been discussed in the literature [2]. Second, there is an increasing body of evidence on the involvement of the immune system. Negative life events, like bullying, have been shown to be related to inflammation in later life [62]. For instance, a Swedish study found increased incidence of autoimmune disorders after trauma [60], and a Norwegian study demonstrated elevated levels of cytokines in patients undergoing treatment for PTSD [64]. The immune system is involved in defence against infection and the in morbidity and mortality in infection, malignancy, autoimmunity and cardiovascular/atherosclerotic disorders; consequently, it is highly important to further explore the reported immune deviations related to psychological trauma.

In terms of treatment for PTSD, several individual trauma-focused treatments such as eye movement desensitization training (EMDR), cognitive behavior therapy (CBT) and prolonged exposure (PE), have been documented to be efficacious, and to have similar effects [6]. However, a substantial portion of patients with PTSD do not improve or have residual symptoms after such specialized treatment Bradley et al. [56]. Moreover, many individuals who have recovered from PTSD symptoms may still have a significantly lower quality of life and reduced level of functioning compared to exposed persons who never developed PTSD (Westphal et al. [68]). Importantly, the effectiveness of the specialized trauma-focused treatments in large community settings has been questioned, as the majority of randomized controlled trials on PTSD have been conducted within military populations and military settings [14], a population that may differ from the population seen in the typical mental health setting.

Taken together, the complex and interwoven picture of somatic and mental health problems seen in a large group of patients in general mental health services may require more, and possibly different, treatment than among individuals with less complex diagnostic pictures. Given the substantial costs of trauma-related conditions at both the individual and societal level, there should be a prime interest in developing methods for helping this group of patients. One of several concerns in the development of such methods should be the feasibility of implementation in ordinary clinical settings. A transdiagnostic approach has been called for [28], as have alternatives to treatment interventions focusing on exposure [50, 57]. As indicated in a literature review, specific factors such as exposure become less important with increasing problem complexity [29]. Emotion regulation together with cognitive behavioral strategies might be useful in more complex posttraumatic suffering [39].

Current treatments for complex posttraumatic disorders have been heavily criticized for overemphasizing the need for phase-based treatment, and thus risking that patients may be delayed in receiving evidence-based treatments [20]. Another disagreement in this field is related to the conceptual understanding of the symptoms. The newest editions of the diagnostic manuals differ in how they conceptualize PTSD as a diagnosis. While the single PTSD diagnosis was expanded in the Diagnostic and Statistical Manual of Mental Disorders, Fifth Edition (DSM-V) (2013), the ICD 11 (2018) proposed two sibling diagnoses: PTSD and complex PTSD or CPTSD [43]. Accordingly, there is a need for more knowledge on how to efficiently help patients receiving treatment in ordinary mental health care to recover from long-lasting and complex posttraumatic symptoms.

Elements of group psychoeducation and psychotherapy have been shown to be promising complements to individual psychotherapy in the treatment of posttraumatic suffering [58]. In addition to the effective delivery of knowledge and understanding of common trauma reactions in a group format, several of the therapeutic factors that typically arise in group settings (e.g., normalization, reduced experience of shame and corrective relational experiences) have been identified as particularly useful for survivors of repeated emotional or relational traumatic experiences [17]. Recent qualitative studies provide examples of healing therapeutic change processes within groups of people with complex interpersonal trauma [13, 61]. Still, research on group-based treatment for posttraumatic disorders has lagged behind the attention given to individual interventions [59]. In routine clinical practice, individual treatment is much more frequent than group-based approaches. A national report showed that within specialized community mental health hospitals in Norway, group-based treatment was given to only 13% of the patients, while the majority (82%) received individual-based therapy for their mental health problems [52]. Consequently, patients with long-lasting posttraumatic reactions are most likely to receive only individual-based therapy approach. Moreover, due to limited resources, very few of these patients will meet with their therapists more often than bi-weekly or, at best, weekly. However, similar to findings from research into depression [18], clinical experience with patients with posttraumatic problems indicates that treatment of some intensity (i.e., more frequent sessions) substantially augments the effect of treatment in trauma survivors. Accordingly, adding a group-based module to individual treatment may be a cost-effective way to intensify treatment both in terms of content (psychoeducation, skill training and group therapeutic factors) and frequency of sessions.

To meet the need for a broad and integrated treatment model for patients with complex PTSD, the Modum Bad Trauma Clinic issued a treatment manual in 2014 named “Tilbake til Nåtid” (“Back to the present” [34];). The group-based treatment approach detailed in this manual uses stabilization and skill training (SST) to increase participants’ affect regulation when exposed to triggers, and to increase their capacity of stabilization or level of tolerance to handle unpleasant reminders. The course, lasting for 2 h per session over 20 weeks, is combined with individual treatment, not specified. The courses have been running at Modum Bad since 2008. A randomized controlled trial has been carried out in this highly specialized setting (results to be published).

Results have been mixed in research on similar treatments. Zlotnic et al. [73] demonstrated a considerable effect in a randomized controlled trial; similarly, a preliminary, non-controlled study in Scotland showed promising results [36]. However, results obtained by Dorrepaal et al. [22] were less clear, with only a non-significant trend towards symptom reduction.

Clinical experience with this particular SST approach is promising, as seen both from two outpatient clinics around Oslo that have implemented the treatment [47] and from our own community mental health hospital, where the course has been completed more than 10 times since 2015. The programme has been well-received by patients and clinicians. In an unpublished evaluation, we collected pre-programme and post-programme data in four questionnaires (Symptom Checklist (SCL-90), Impact of Event Scale (IES), Dissociative Experience Scale (DES) and Somatic Experience Scale (SES)) in the first 33 participants, of whom 18 (56%) completed data collection both at the start and the end of the course. We identified a significant decrease in symptom level. The drop-out rate was acceptable (approximately 20%) and was mostly due to change in occupational or educational status. Overall, feedback on the course, as given by participants and individual therapists, was positive. The systematic evaluations and feedback given by participants at the end of the courses has guided the development of the present research project.

In sum, a large group of patients in mental health care present with complex and severe trauma-related problems. The individual treatment models currently available for this group of patients may not sufficiently address the full range of their suffering and may, moreover, be challenging to implement. Clinical experience and preliminary evaluations indicate that a newly developed group approach, SST, might be a helpful addition to individual treatment. The approach has yet to be tested with a randomized controlled design in an ordinary outpatient setting. The present research project will evaluate the effect of the SST course on patients’ process of recovery. Outcomes will also include broader measures of impairment and general somatic and mental health measures, and mediating therapeutic factors will be examined.

## Methods

### Aims and research questions

The main aim of this study is to compare the effects of conventional treatment alone (treatment as usual (TAU)) to the combination of a group-based SST intervention with individual treatment (intervention group, SST) in outpatients with long-term posttraumatic reactions receiving health services in a community mental health hospital.

The main hypothesis is that adding a group-based SST intervention will increase experienced personal recovery more effectively than conventional, mostly individual treatment. Several secondary research questions and hypotheses will be explored, such as the following. Will the combined treatment be superior to individual treatment alone in terms of somatic and psychological health, functional impairment, and general wellbeing, use of health services, pharmacological treatment and occupational status following treatment? Will changes in posttraumatic cognitions during treatment mediate the outcome? What characterizes the immune and endocrine systems of service users of general mental health care who seek treatment for longstanding posttraumatic reactions?

### Trial design

**Fig. 1:**
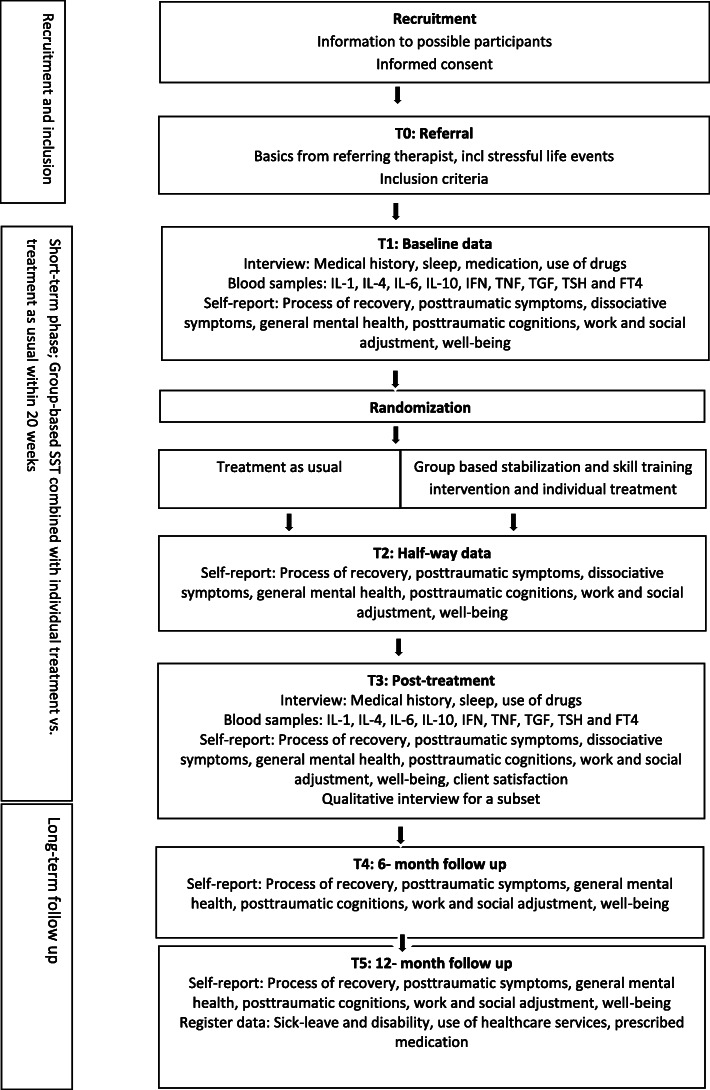
Diagram of flow of participants. TSH, thyroid stimulating hormone; FT4, free thyroxine; SST, stabilization and skill training

**Fig. 2:**
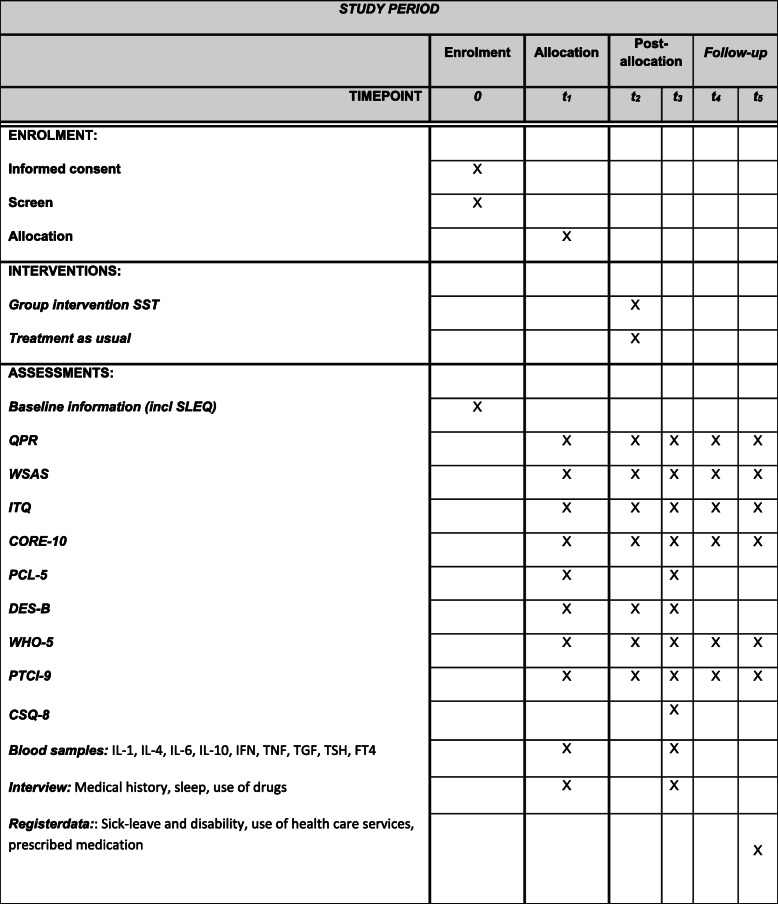
Schedule of enrolment, interventions and assessments. SLEQ, Stressful Life Events Screening Questionnaire; QPR, Questionnaire Process of Recovery; ITQ, International Trauma Questionnaire; PCL-5, Posttraumatic Checklist; DES-B, Brief Dissociative Experience Scale; CORE-10, Clinical Outcome Routine Evaluation; WSAS, Work and Social Adjustment Scale; WHO-5, World Health Organization Well-Being Index; PCI-9, Posttraumatic Cognition Inventory; CSQ, Client Satisfaction Questionnaire; SST, stabilization and skill training; IFN, interferon; TGF, transforming growth factor; TSH, thyroid stimulation hormone; FT4, free thyroxine

The study is a single-site, non-blinded, randomized controlled trial with two arms, TAU or a combination of a group-based SST intervention with individual treatment (intervention group, SST). As this is a highly naturalistic study, blinding of participants, therapists and researchers to the results of the randomization is not feasible. See Fig. 1 for details of the study design. The protocol has been prepared in accordance with the Standard protocol items: recommendation for interventional trials (SPIRIT) checklist (see Additional file 1) and SPIRIT figure (see Fig. 2).

### Study setting

The study will be conducted at the Tiller community mental health centre; this is a department of St. Olavs Hospital, which is the university hospital in Trondheim, Norway. The department is a specialized, secondary community mental health hospital for adults and serves approximately 85,000 inhabitants in two municipalities, including both urban and rural areas. Enrolment to the study started in March 2019 and the data collection is expected to be finished by the end of 2023.

### Eligibility and inclusion criteria

Adults (age ≥ 18 years) who (1) are already receiving treatment in ordinary outpatient clinics, (2) have been exposed or witnessed traumatic event(s) defined as “one or several extremely threatening or horrific events or series of events or situations being of such a character which is likely to overwhelming distress in almost anyone in a similar situation”, in line with criteria for the definition of PTSD and CPTSD in ICD 11 [71], (3) present with posttraumatic reactions such as hyperarousal, avoidance, intrusions, emotional dysregulation or interpersonal difficulties, (4) have symptom duration of minimum of 6 months and (5) understand and speak Norwegian to the extent that they are able to participate in a SST group. Exclusion criteria are active psychotic symptoms, substance abuse, high suicidal risk as assessed by the individual therapist or previous participation in the SST group.

### Recruitment procedures

A total of 160 patients (age ≥ 18 years) will be recruited from the population of patients already receiving treatment in ordinary outpatient clinics. Prospective participants are first informed about the project by their individual therapist. Those who express interest in participating in the study meet with the group therapists together with their individual therapist to receive additional information. Prospective participants then sign an informed consent form and return it by mail or to the reception desk at the clinic. Next, the individual therapist completes a referral questionnaire, which includes basic information including information about previous traumatic incidents based on a standardized checklist, The Stressful Life Events Screening Questionnaire Revised (SLESQ) [30]. The referral documentation also includes information on diagnoses, demographics, current posttraumatic reactions, medication and substance use. In accordance with routine practice at the clinic, the individual therapist performs the diagnostic evaluations based on ICD-10 criteria, and the diagnosis is confirmed in a consensus meeting at the outpatient clinic with participation of at least one specialist in clinical psychology or psychiatry.

All referrals are initially screened for eligibility by a team of group therapists. If the criteria are fulfilled, the participant is added to a waiting list. When sufficient participants are recruited to fill a cluster (approximately 18–20; 9–10 in the intervention group and 9–10 in the TAU group) participants are contacted and invited for initial assessment and randomization. In order to achieve an adequate sample size, therapists will be regularly be reminded of the research project and encouraged to refer eligible participants to it.

### Assessment

Prior to randomization, participants undergo a baseline assessment package (T1). This includes self-reports, a short interview and collection of blood samples. Subsequently, self-report data are collected in both conditions when the group intervention reaches the halfway mark approximately 4 months after baseline (T2; mid-treatment), approximately 4 weeks after the course has ended i.e., 8 months after baseline (T3; post-treatment), 6 months after the end of the group intervention i.e., 13 months after baseline (T4; follow up) and 12 months after the end of the intervention i.e., 19 months after baseline (T5; follow up). The collection of blood samples and a short interview will be repeated at post-treatment and the individual therapist will answer a set of questions at the end of the intervention (T3).

Questionnaires are sent to participants by mail and returned either by mail or by personal delivery to the clinic. Participants who fail to return the questionnaires are contacted a maximum of twice, either by sending another copy of the questionnaire and/or by phone.

### Interventions

Participants in both arms of the study continue to receive conventional individual treatment from the therapist to whom they are already allocated. As the study is carried out in a natural setting, the content of this conventional therapy varies and is not standardized in any manner, but information on the content of individual treatment is collected from therapists at post-treatment (T3). The outpatient clinic offers medical and psychological treatment for mental illness. All patients have an individual therapist, in most cases a psychologist or psychiatrist. If the primary therapist is not a psychiatrist or a senior psychologist, one such will be involved in the treatment as supervisor and counsellor. All patients are discussed in treatment teams that include specialists and medical doctors.

Participants in the intervention group will also receive a group-based SST intervention based on the manual “Tilbake til Nåtid” (“Back to the present” [34];), which was developed by clinicians at Modum Bad’s Trauma Clinic, a highly specialized national trauma clinic. The manual is based on work by Boon and colleagues [9] and has been changed and adapted by the Norwegian authors. The manual is intended for use by both the participants and the therapist; it teaches psychoeducation and skills relevant to people with long-lasting trauma reactions. It is based on several theoretical models and also covers everyday routines and knowledge related to somatic and mental aspects. The manual also encourages reflections and discussions among the participants on how to regulate difficult affects and interpersonal difficulties that may arise as a result of triggers from previous traumatic experiences.

The manual is organized into 20 chapters and covers the following topics: (1) introduction and motivation, (2) window of tolerance, (3) understanding PTSD, (4) complex traumatization, (5) dissociation, (6) understanding trauma memories and triggers, (7) coping with triggers, (8) mindfulness, (9) bodily reactions, (10) summary and review of content so far, (11) establishing a daily structure, (12) safe sleep, (13) trauma-related cognitions, (14) reflective skills, (15) understanding emotions, (16) self-compassion, (17) coping with anger, (18) fear of relations, (19) assertiveness and boundaries and (20) evaluation and goodbye for now.

The course runs for 20 weeks, and has a fixed rhythm for every session. Each session lasts for 2 h and includes a short break of 15 min. The first hour is a review of the chapter from the previous week, with participants’ reflections and experiences linked to that topic, and feedback on homework and skill training. The second hour is devoted to a new chapter. Based on our previous experiences and feedback from the group participants in the course, participants will be invited to attend a meeting approximately 2 months after completing the course to consolidate their learning and exchange feedback with the other participants and therapists.

Although group sessions are structured, participants are encouraged to share their everyday experiences with coping and handling troublesome episodes related to posttraumatic suffering. The rules and regulations of the group dictate that participants are not permitted to share traumatic or highly upsetting material with the other patients. Instead, participants are encouraged to address any issues that cannot be handled within the group setting with their individual therapist.

Each group has 8–10 participants and the groups are led by two trained therapists from a pool of several professionals who have experience with the treatment manual. Professionals include, to date, two psychomotor physiotherapists, two senior clinical social workers, four psychiatric nurses and three senior clinical psychologists. Some additional clinicians will be recruited as group leaders during the project. New group therapists are paired with experienced therapists in accordance with a master-apprentice model.

The RCT is integrated into routine care; as no serious adverse treatment effects are expected in either condition, there will be no special criteria for discontinuing or modifying allocated interventions. Participants in both conditions will have the opportunity to continue individual treatment after the trial if necessary and appropriate.

### Outcomes

The outcomes are self-report inventories, clinical assessments and register-based data that will be used to detect potential changes in a variety of outcomes.

### Primary outcome

The primary outcome is change in the personal recovery experienced from pre-treatment to post-treatment and follow up measured by the Questionnaire about Process of Recovery (QPR) [49]. The scale was originally developed in collaboration with service users, and has been widely used to capture peoples’ accounts of recovery from severe mental illness. Participants are asked about aspects of recovery that are meaningful and relevant to a broad range of patients. In line with the definition of personal recovery, the items capture “a process of changing one’s attitudes, values, feelings, goals, skills, and/or roles. It is a way of living satisfying, hopeful and countering life even with limitations caused by illness” [69]. The 15-item version has been recommended for use both in routine clinical work and in research, and it is scored on a 5-point scale from 0 = disagree strongly to 4 = agree strongly). The psychometric properties in terms of internal consistency, test re-test reliability and convergent validity were found to be high [42].

### Secondary outcomes

Perceived functional impairment associated with mental health problems is assessed using The Work and Social Adjustment Scale (WSAS [48];), a self-report questionnaire. The scale has five items covering the following dimensions; influence on work, home management, social leisure activities, private leisure activities and relationships with others. The items are scored from 0 to 8, with a total score from minimum 0 to maximum 40, with lower scores indicating better adjustment. The scale has been found to have high internal reliability and sensitivity to treatment effects and has been recognized as an additional outcome measure alongside traditional symptom scales [72]. The scale was recently found to be a reliable, unidimensional and gender-invariant measure, sensitive to change among Norwegian outpatients with interpersonal problems [53].

Symptoms of PTSD and of complex PTSD (CPTSD), as presented in ICD-11, are measured with the newly developed self-report questionnaire the International Trauma Questionnaire (ITQ [15];). The ITQ contains 12 items: 6 covering the three clusters of PTSD (re-experiencing of the trauma in the here and now, avoidance of traumatic reminders and persistent sense of current threat that is manifested by arousal and hypervigilance), and 6 reflecting the three clusters of disturbances in self-organization (DSO) in CPTSD (affective dysregulation, negative self-concept and disturbances in relationships). Items are scored on a Likert scale from 0 (not at all) to 4 (extremely) to indicate how much the participant has been bothered by each problem during the past month. A PTSD diagnosis requires a score of ≥ 2 (moderately) for at least one symptom in each of the three clusters plus endorsement of functional impairment associated with these symptoms. For a likely CPTSD diagnosis an additional score of at least ≥ 2 (moderately) is needed for at least one of the symptoms in each of the three DSO clusters. The provisional initial English version of the ITQ demonstrated satisfactory psychometric properties [11, 37, 38]. The Norwegian version was translated at Modum Bad’s Trauma Clinic, and we made some minor adaptions to the final version recently provided to us by the authors of the scale.

General psychological distress is assessed using the Clinical Outcome in Routine Evaluation (CORE-10 [4];), a brief measure of capturing general mental health issues such as anxiety (two items), depression (two items), trauma (one item), physical problems (one item) functioning (three items) and risk to self (one item). It has excellent psychometric properties [4]. Participants are asked to report how often they have experienced the symptoms during the past week on a 5-point Likert scale from 1 (never) to 5 (almost all the time).

Additional trauma-related symptoms are assessed using the Posttraumatic Checklist (PCL-5) [66], a 20-item self-report measure of PTSD based on the criteria from DSM-V. Items are scored on a 5-point scale and range from “not at all” to “extremely”. The scale was found to have adequate psychometric properties [7] and has been translated into Norwegian by the Norwegian Centre for Violence and Traumatic Stress Studies (NKVTS). Moreover, the Brief Dissociative Experience Scale (DE-B), a 9-item short form of the Dissociative Experience Scale-II [12], is included to assess dissociative experiences with regard to memory, identity, awareness and cognition.

Quality of life is assessed using the WHO Five Well-Being Index (WHO-5) [5], one of the most widely used measures of subjective psychological well-being that is also a measure of depression. The scale consists of five simple and non-invasive questions about participants’ well-being experienced during the past 2 weeks, and items are rated on a 6-point scale from 0 (never) to 5 (all the time). A recent review demonstrated that the questionnaire has excellent psychometric properties [65].

Negative and dysfunctional posttraumatic cognitions are assessed using the Posttraumatic Cognition Inventory (PTCI-9) [67], a shortened version of the original 33-item PTCI scale [26]. The scale captures negative cognitions about self, negative cognitions about the world and self-blame. The nine items are rated on a Likert scale ranging from 1 (totally disagree) to 7 (totally agree). Good psychometric properties have been shown for the original and for the newly developed short version [67].

Treatment satisfaction in both conditions is assessed using the Client Satisfaction Questionnaire (CSQ-8 [3];), a shorter version of the original 18-item version [41]. The measure yields a continuous score on a single factor of general satisfaction. This is derived from eight questions about the quality of service, the kind of service, patients’ outcomes and overall satisfaction. The scale is rated on a 4-point response scale and it has been used extensively in research.

Somatic health is assessed in interviews, using a semi-structured interview guide developed for this study; the guide elicits information about somatic illness, sleep problems, diagnoses and treatment in specialized health care. Additionally, height and weight are recorded for calculation of body mass index (BMI). Blood samples will be collected for measurement of changes in the inflammatory markers, interleukin (IL-1, IL-4, IL-6, IL-10), interferon (IFN), tumor necrosis factor (TNF) and transforming growth factor (TGF) from pre-treatment to post-treatment; plasma will be frozen for subsequent analyses of cytokines using the multiplex ELISA technique. Thyrotropin releasing hormone (TRH) and free thyroxine (FT4) will be measured in blood to assess changes in endocrine markers. Register data from three sources will be collected. First, data on use of mental health services 12 months before randomization and at least 12 months after the end of the group intervention will be extracted from the National Patient Register (NPR), which regularly receives data from all health authorities in Norway on treatment episodes in general hospitals, mental health services and substance abuse services. Data will include type of clinical unit, time of admission, length of stay in inpatient units, consultations in outpatient units and mobile teams and diagnoses. Second, data on occupational status will be derived from the nationwide electronic register (NAV/SSB). Extracted data will include welfare or occupational status, number and length of episodes of sick-leave or disability leave. Third, data on use of pharmacological treatment will be collected from hospital records and the national register for pharmacological treatment.

Additionally, participants in two or three of the clusters (both arms; TAU and SST), will be invited to participate in a semi-structured interview to share their experiences of treatment during the last 8 months (since inclusion in the project).

### Fidelity

The manual has yet to be validated and there are no measures of fidelity available to date. To ensure fidelity and adherence to the group intervention, the manual will be actively used in each group session. The manual will also guide the group discussion/s of how to regulate trauma-related affects and interpersonal difficulties relevant to the weekly chapter. Using a checklist, the group leaders rate the presented topics in order to ensure that the core components are delivered in all the courses. Also, when recruiting new group therapists, the new therapists are paired with experienced therapists in accordance with a master-apprentice model.

### Sample size

To the best of our knowledge, there are no established parameters for reliable and/or clinically significant change in the main outcome measure of this study, the QPR-15. We determined the minimal effect size that would be clinically meaningful to be Cohen’s *d* = 0.5; given the costs of implementing SST groups, a smaller effect size would be of little interest to the clinic. Given α = 0.05 and statistical power of 0.80, we calculated that the trial requires a total of 126 participants, 63 in each arm. Accounting for drop-out, which was 20% in the pilot testing of the group intervention, we plan to enrol a total of 160 participants, 80 in each arm. Several of our secondary outcome measures, e.g. the Work and Adjustment Scale (WSAS) are widely used. Three studies utilizing the WSAS comparing new treatments with TAU identified a mean change between the intervention group and TAU from 4.04 [54], 4.5 [55] and 4.6 [21]. These give a mean score of 4.38. The mean standard deviation for pre-treatment and post-treatment scores was 8.5. A power calculation with α = 0.05 and statistical power of 0.80 requires a total sample of 120, which is in line with our first sample size calculation. The estimations are further in line with general recommendations of 75 persons in each arm for studies with two active arms [1].

### Randomization

Randomization to the intervention group or TAU will be performed through a web-based system developed and administered by the Unit of Applied Clinical Research, Norwegian University of Science and Technology (NTNU), Trondheim, Norway. Participants will be randomized sequentially, in blocks of variable size (i.e., seven to nine group participants) to ensure even allocation. Participants will be stratified in order to ensure the gender ratio.

### Data management

The Data Protection Official at St. Olavs Hospital has approved the data management plan for this study. Data from several sources are collected: (1) therapist referral sheet with background information, (2) blood samples, (3) baseline interviews of participants, (4) participant pen-and-paper self-report and (5) register data. To secure anonymity, participants are given a non-identifiable study ID at randomization. This ID is used throughout the study for data storage and analysis.

Treatment in both arms is delivered in accordance with routines at the clinic and the intervention under investigation is considered low risk. As there are no anticipated detrimental effects or concerns over patient safety associated with this study, a data monitoring committee, interim analyses or stopping guidelines are not considered necessary. As part of routine care, all potential adverse events will be taken care of by the participant’s individual therapist and will, in accordance with hospital policies, be reported to the head of the clinic.

### Statistical methods

The efficacy of the group intervention will be evaluated on an intention-to-treat basis comparing the two arms (SST and TAU). The main efficacy analysis will pertain to the data obtained at post-treatment and 8 months follow up, using *t* tests or mixed models to test for differences in changes in the primary outcome scores from pre-treatment to post-treatment. Baseline characteristics will be tabulated by treatment modality. Per-protocol analyses will also be run.

We will use explorative mediation analyses to investigate whether posttraumatic cognitions influence the outcome measures. We will also consider longitudinal analysis of individual growth curves or analysis of mixed linear models as appropriate.

### Qualitative analyses

Interview data will be analyzed according to consensual qualitative research (CQR) [33], which is a structured, inductive, consensus-based, integrative analytic methodology.

### Plan for dissemination of results

Results will be disseminated in articles published in peer-reviewed journals and conference presentations at national and international conferences. Dissemination to the general public will be in concordance with hospital policies, including seminars for healthcare workers and service users.

## Discussion

The aim of this trial is to test the effect of combining a group-based stabilization and skill-training intervention with individual treatment in outpatients with long-term posttraumatic reactions, who are receiving health services in a community mental health hospital. Existing individual treatment approaches may not be sufficient to alleviate the considerable mental and somatic health problems of this large group of patients. Group treatment is cost effective and allows for change in processes that are difficult to achieve with individual treatment alone. Based on clinical experience, we consider this novel approach to be highly promising, but it has not previously been tested in an ordinary mental health setting.

As discussed in the introduction, research on group treatment in complex trauma-related mental health conditions is scarce. The current trial has several advantages over other studies. First, the naturalistic approach ensures that results may easily guide further implementation in the clinic, depending on the results. Second, the longitudinal study design and repeated measures allow us to observe the development of outcomes during the intervention. Third, the relatively long follow up allows us to observe the possible effectiveness over quite some time. Fourth, in contrast to the sole focus on either quantitative or qualitative outcomes in previous research on similar interventions, the current study will adopt a mixed methods approach. Combining data from several sources, including self-reported data on somatic and mental health outcomes, objective measures of somatic health, qualitative interviews and governmental register data on employment status and use of health care services allows for a thorough in-depth understanding of the impact of the intervention for the individual and for society.

The main limitation of this study is that it does not compare the combined group and individual treatment to any gold standard procedure for treatment of PTSD, such as CBT or EMDR. Nor does it provide a pure test of the treatment manual as such, as it will be impossible to separate the impact of the individual treatment from the impact of group treatment on participants in the intervention condition. If the combined treatment is found superior to individual treatment alone, further studies will be needed to compare combined treatment to a gold standard treatment, including studies featuring a control condition with a comparable treatment intensity (e.g., a combined individual and group treatment control condition).

Another concern is that the study is limited to a single site. A multi-centre trial may also be needed to test the intervention in additional settings. However, testing the intervention first in a single centre might have some benefits, as the number of variables will be reduced.

The limitations notwithstanding, the strength of the study lies in its pragmatic and clinically relevant research questions and in the highly naturalistic design, which substantially increases the generalizability of results. This study will be of interest to those searching for more effective ways to treat a large group of patients in ordinary mental health care for difficulties resulting from compound strain in the past. If a time-limited group-based approach is found beneficial in a broad range of outcome measures, this may have important implications for the organization and development of mental healthcare services.

### Registration and trial status

The reporting of the results will follow the 2017 Consolidated standards of reporting trials (CONSORT) guidelines for reporting a randomized trial assessing nonpharmacological treatments. The protocol is developed according to the CONSORT Standard protocol items: recommendations for interventional trials (SPIRIT 2013 checklist: see Additional file 1). The trial was registered within ClinicalTrials.gov (NCT03887559) on 25 March 2019. Recruitment began on 9 April 2019. Recruitment will be completed by approximately 31 March 2023.

## Supplementary information

**Additional file 1.** SPIRIT 2013 checklist.

## Data Availability

The full protocol is made publicly available through registration in Clinical Trials.gov (NCT03887559) and this publication. At present, we have made no specific plan for sharing participant data. Any data required to support the protocol can be supplied on reasonable request. In the case of significant changes, the protocol will be updated in the clinical trial registry and reported to the Regional Committee for Medical Research Ethics.

## Abbreviations

CORE: The Clinical Outcome in Routine Evaluation
CPTSD: Complex posttraumatic disorder
CSQ: The Client Satisfaction Questionnaire
DES-B: The Brief Dissociative Experience Scale
ELISA: Enzyme-linked immunosorbent assay
FT4: Free thyroxine
ICD-11: International Classification of Diseases 11th Revision
IL: Interleukin
IFN: Interferon
ITQ: The International Trauma Questionnaire
PCL: The Posttraumatic Checklist
PTCI: Posttraumatic Cognition Inventory PTCI
PTSD: Posttraumatic Stress Disorder
QPR: Process of Recovery Questionnaire
SST: Stabilization and skill training
TGF: Transforming growth factor
TNF: Tumor necrosis factor
TRH: Thyrotropin releasing hormone
TSH: Thryoid stimulating hormone
WHO-5: The WHO Five Well-Being Index
WSAS: The Work and Social Adjustment Scale
